# Docking Studies on Potential Mechanisms for Decreasing Insulin Resistance by the Tangzhiqing Herbal Formula

**DOI:** 10.1155/2020/1057648

**Published:** 2020-10-09

**Authors:** Jia Hao, Lifeng Han, Yi Zhang, Tao Wang

**Affiliations:** ^1^Tianjin State Key Laboratory of Modern Chinese Medicine, Tianjin University of Traditional Chinese Medicine, 10 Poyanghu Road, West Area, Tuanbo New Town, Jinghai District, Tianjin 301617, China; ^2^Tianjin Key Laboratory of TCM Chemistry and Analysis, Institute of Traditional Chinese Medicine, Tianjin University of Traditional Chinese Medicine, 10 Poyanghu Road, West Area, Tuanbo New Town, Jinghai District, Tianjin 301617, China

## Abstract

Insulin resistance (IR) is considered as one of the principal pathways of type 2 diabetes mellitus pathogenesis and is associated with a series of abnormal signaling pathways. Tangzhiqing (TZQ) herbal formula is a well-known antidiabetic traditional Chinese medicine and has been used to treat type 2 diabetes mellitus and prediabetes for many years in China. We selected 13 natural products as representative compounds of the main active components in TZQ to investigate the interaction of these natural products with key signal proteins associated with IR using two different docking calculations. Salvianolic acids A and C (phenolic acids from *Salvia miltiorrhiza*), rutin (a flavonoid from *Morus alba*), paeoniflorin (a saponin from *Paeonia lactiflora*), and quercitrin (a flavonoid from *Crataegus pinnatifida*) showed great docking abilities towards multiple target proteins. These results have contributed to a clearer understanding regarding the regulation mechanism of TZQ on IR and have provided direction for further pharmacological studies.

## 1. Introduction

Insulin resistance (IR) refers to a pathological status in which there is decreased biological regulation of insulin. Its clinical manifestation is the decrease in insulin sensitivity and the impairment of glucose utilization in peripheral tissues. Epidemiological studies suggest that IR is the initial occurrence that results in numerous sequelae such as glucose intolerance, dyslipidemia, hypertension, heart disease, and cerebrovascular disease [1–3]. IR is considered as one of the principal events, resulting in the pathogenesis of type 2 diabetes mellitus. Therefore, there has been intense research on how to prevent and alleviate insulin resistance with the goal of preventing the development of diabetes.

**Figure 1:**
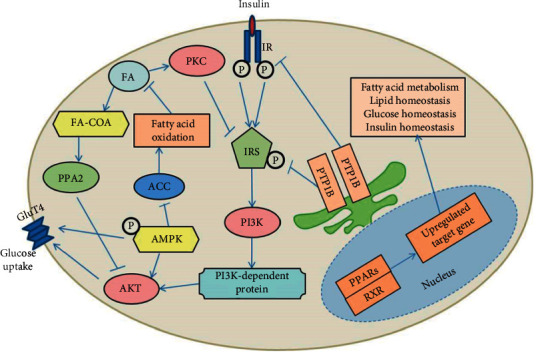
Critical signal pathways associated with IR.

IR is a complex metabolic abnormality with a highly complex pathogenesis consisting of the dysfunction of insulin secretion, insulin receptor-related gene changes, and a series of abnormal downstream signaling pathways [4, 5]. As shown in Figure 1, when insulin binds to an insulin receptor (InsR), insulin receptor substrate (IRS) is translocated to the membrane and further phosphorylated by the insulin receptor. This process stimulates the binding of IRS to the regulatory region p85 in phosphoinositide 3-kinase (PI3K), leading to the activation of PI3K. PI3K-dependent proteins such as PDK-1 are subsequently activated. Subsequent phosphorylation of serine/threonine kinase AKT/protein kinase B leads to the translocation of glucose transporter 4 (GluT4) from glucose transport vesicles to the cell membrane, which ultimately promotes glucose uptake and utilization.

It has been established that dysfunction and negative regulation of the insulin-mediated signaling pathway can affect insulin activity, resulting in insulin resistance. When tyrosine phosphatase 1b (PTP1B) dephosphorylates insulin receptors, this action can lead to insulin resistance [6]. Fatty acid accumulation, inflammation, and oxidative stress can also lead to insulin resistance by the activation of a series of signaling proteins with negative regulatory effects on the insulin-mediated signaling pathway, such as PKC, protein phosphatase A2 (PPA2), and C-Jun N-terminal kinase (JNK) [7–9]. Therefore, many critical pathways in lipid metabolism also play an important role in relieving insulin resistance. All these key proteins are important drug development targets for decreasing insulin resistance. Available therapeutic strategies for insulin resistance are limited by problems of side effects and insufficient efficacy. Thus, there is continuous ongoing research to formulate efficacious medications for insulin resistance. It has been demonstrated that a combination of drugs with diverse therapeutic targets has good clinical efficacy in decreasing insulin resistance.

Traditional Chinese medicine (TCM) has a long history of use in the treatment of diabetes mellitus. Its clinical efficacy is associated with the synergistic effect of several small molecular drugs with multiple targets. This therapeutic strategy is very effective for the clinical treatment of chronic diseases with complex pathological mechanisms, such as insulin resistance. Tangzhiqing (TZQ) is a well-known antidiabetic formula containing white mulberry leaf (*Morus alba*), lotus leaf (*Nelumbo nucifera*), Chinese sage root/Danshen (*Salvia miltiorrhiza*), hawthorn leaf (*Crataegus pinnatifida*), and red peony root (*Paeonia lactiflora*) and has been used to treat type 2 diabetes mellitus and prediabetes for many years in China [10]. Based on our group's previous work, it has been proved that TZQ can decrease insulin resistance symptoms, reduce fasting and postprandial blood sugar levels, and regulate blood lipids in model rats [11–14]. The main active components of this prescription were identified as *Paeonia lactiflora* saponins, *Nelumbo nucifera* alkaloids, *Nelumbo nucifera* flavonoids, *Morus alba* alkaloids, *Morus alba* flavonoids, *Morus alba* polysaccharide, *Salvia miltiorrhiza* phenolic acids, and *Crataegus pinnatifida* flavonoids [15–17].

In this study, we selected 13 natural products as representative compounds of the main active components in TZQ to investigate the interaction of these natural products with key signal proteins associated with insulin resistance using molecular docking methods. As a continuing study, this work will pave the way for further explorations towards the mechanism of TZQ antidiabetic activity.

## 2. Experimental

### 2.1. Materials and Methods

#### 2.1.1. Ligand Preparation

We selected 13 natural products as representative compounds of the active components in TZQ (Table 1; the chemical structures are listed in the Supporting Information (available (here)). We chose rutin and quercetin as representatives of flavonoids: 1-deoxynojirimycin and nuciferine as representatives of alkaloids; salvianolic acid A, salvianolic acid B, salvianolic acid C, danshensu, and rosmarinic acid as representatives of phenolic acids; tanshinone IIa, cryptotanshinone, and dihydrotanshinone I as representatives of tanshinones; and paeoniflorin as a representative of saponins. The structures of these natural products were constructed using ChemBioDraw software and were optimized by dreiding-like forcefield and CHARMm forcefield separately. The prepared ligand function was used for conformation generation.

#### 2.1.2. Target Protein Preparation

Phosphatidylinositol 3-kinase (PI3K and PDB ID: 4WAF) [18], protein kinase B (AKT and PDB ID: 3OCB) [19], tyrosine phosphatase 1b (PTP1B and PDB ID: 2QBP) [20], adenosine monophosphate-activated protein kinase (AMPK and PDB ID: 4CFE) [21], peroxisome proliferator-activated receptor *α* (PPAR-*α* and PDB ID: 3VI8) [22], peroxisome proliferator-activated receptor *δ* (PPAR-*δ* and PDB ID: 3TKM) [23], and peroxisome proliferator-activated receptor *γ* (PPAR-*γ* and PDB ID: 1K74) [24] were selected as key signal proteins. The three-dimensional crystal structures of their corresponding protein-active complexes were obtained from the RCSB Protein Data Bank (PDB) library. Relevant receptors and ligands were defined. The ligand and water molecules in the crystal structures were deleted, and polar hydrogen was added to the remaining clean proteins. Then, all of the proteins were optimized using the clean protein and prepare protein function.

#### 2.1.3. Docking Study

**Figure 2:**
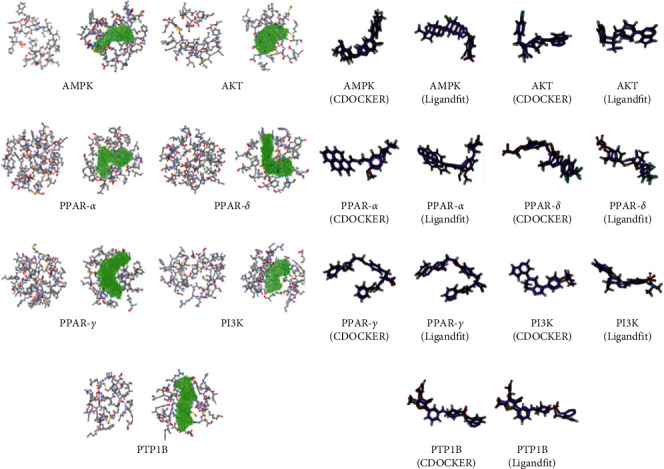
Docking pocket models and redocking results (the actual conformations are showed as purple).

The docking calculation process was accomplished by CDOCKER and the Ligandfit procedure for the protein-ligand interaction module in the DS software. CDOCKER and Ligandfit are typical semiflexible docking methods, both of which use a rigid receptor and a set of multiconformational ligands [25, 26]. The difference between these two methods is that the former is a CHARMm-based docking algorithm, while the latter is a shape-directed docking algorithm. The docking score was evaluated by using the -CDOCKER INTERACTION ENERGY score (CDOCKER) and -PLP score (Ligandfit) separately. The redocking process was performed to validate whether the binding pocket model and docking parameters were appropriate for the followed docking calculation. The root-mean-square difference (RMSD) values of the optimal conformation between the actual conformation and virtual docking conformation starting from a random conformation of the original ligand are listed in Table 2. All of the models can reproduce the actual conformation of the ligand in the crystal structure through the virtual docking process and can be used for subsequent virtual screening studies (Figure 2).

### 2.2. Results and Discussion

Structure-based virtual screening has been considered as an efficient strategy in drug target identification, and various docking algorithms have greatly increased the efficiency of drug discovery [27]. With the continuous development of proteomics, structural biology, and other technologies, an increasing number of three-dimensional structures of biological macromolecules and their active complexes have been resolved, forming rich and complete database resources that facilitate the rapid screening of active lead compounds.

Phosphatidylinositol 3-kinase (PI3K) is a heterodimer composed of regulatory subunit p85 and catalytic subunit p110, with dual activities of phosphatidylinositol kinase and Ser/Thr protein kinase. Dysfunction in either of these two subunits could lead to disorders in glucose and lipid metabolism [28, 29]. The binding of the SH2 domain in regulatory subunit p85 to phosphorylated tyrosine residues can activate catalytic subunit p110, leading to downstream signal transduction. Protein kinase B (called AKT) is an important downstream target protein of the PI3K signaling pathway and is composed of the N-terminal pleckstrin homology domain, intermediate catalytic domain, and C-terminal regulatory domain. The PI3K/AKT pathway plays a crucial role in the insulin-mediated cascade signaling pathway [30, 31].

**Figure 3:**
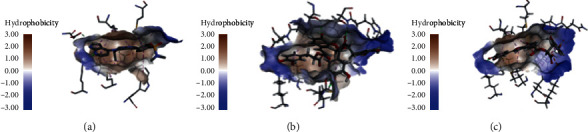
Interactions between PI3K binding site and ligands: (a) initial ligand; (b) top hit of CDOCKER (salvianolic acid B); (c) top hit of Ligandfit (salvianolic acid C).

The 13 selected representative compounds of active components in TZQ were evaluated for their docking abilities towards PI3K and AKT by the CDOCKER and Ligandfit methods, respectively. As shown in Table 3, only paeoniflorin could not dock to the PI3K binding site, and the other natural products exhibited different interaction abilities with PI3K by the CDOCKER calculation procedure, while paeoniflorin obtained a moderate docking score in the Ligandfit calculation procedure. This result may be due to the different adaptabilities of the two algorithms. Nuciferine, 1-deoxynojirimycin, and Danshensu failed to dock with the PI3K binding site during the Ligandfit calculation procedure. Similarly, very low scores were assigned to these three compounds from the CDOCKER calculation procedure. The highest scoring compounds of the two algorithms were the same: salvianolic acids A, B, and C from *Salvia miltiorrhiza* and rutin from *Morus alba*. The top hit compounds shared the same hydrogen bonds interactions and hydrophobic interactions with the key amino acid residues between the initial ligand (3K6). As shown in Figure 3, the key amino acid residues of PI3K binding site were Lys802, Trp 780, Met 800, Ile848, Val 850, Ile 932, and Met 922. According to CDOCKER calculation result, the interactions between the top hit (salvianolic acid B) and the binding site contained hydrogen bonds interaction with Lys802, Val 851, Ser 773, and Thr 856 and hydrophobic interactions with Ile848, Val 850, Met772, and Ile 932. According to Ligandfit calculation result, the interactions between the top hit (salvianolic acid C) and the binding site contained hydrogen bonds interaction with Lys802, Val 851, Asn853, and Ser 854 and hydrophobic interactions with Trp 780, Met 800, Ile848, Val 850, and Ile 932.

As for the docking calculation studies with AKT, the trend of the docking scores between the two algorithms was consistent. The highest scoring compounds of the two algorithms were identical, and they were salvianolic acids A, B, and C, rosemary acid from *Salvia miltiorrhiza*, and paeoniflorin from *Paeonia lactiflora*.

**Figure 4:**
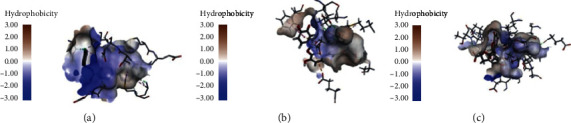
Interactions between AKT binding site and ligands: (a) initial ligand; (b) top hit of CDOCKER (salvianolic acid C); (c) top hit of Ligandfit (salvianolic acid B).

The top hit compounds shared part of the same hydrogen bond interactions and hydrophobic interactions with the key amino acid residues between the initial ligand (XM11). As shown in Figure 4, the key amino acid residues of AKT binding site were Ala230, Glu278, Asp292, Val164, Lys179, Met281, Thr291, and Ala177. According to the CDOCKER calculation result, the interactions between the top hit (salvianolic acid C) and the binding site contained hydrogen bonds interactions with Lys179, Ala230, Asp292, and Glu198 and hydrophobic interactions with Phe161, Val164, Lys179, and Met227. According to the Ligandfit calculation result, the interactions between the top hit (salvianolic acid B) and the binding site contained hydrogen bonds interactions with Ala230, Glu228, Glu234, and Glu 191 and hydrophobic interactions with Gly157, Phe161, Val164, Ala177, and Leu158.

Protein tyrosine phosphorylase 1B (PTP1B) is an intracellular tyrosine phosphorylase that plays an important role in glucose homeostasis, energy metabolism, and other physiological processes. PTP1B consists of a catalytic domain and C-terminal residue. The former domain contains the substrate binding site P-loop and the regulatory site WPD loop, while the latter domain acts as a linkage between PTP1B and the endoplasmic reticulum's cytoplasmic surface. PTP1B negatively regulates the insulin signaling pathway by dephosphorylating the key tyrosine residues of InsR and IRS, leading to decreased insulin sensitivity and increased insulin resistance [32, 33]. Therefore, it is an important and effective drug target for diabetes and obesity.

**Figure 5:**
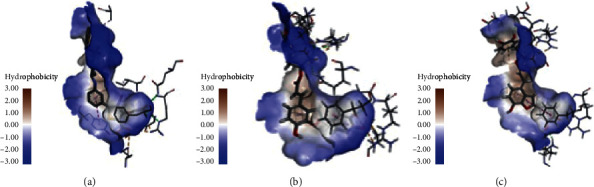
Interactions between PTP1B binding site and ligands: (a) initial ligand; (b) top hit of CDOCKER (salvianolic acid C); (c) top hit of Ligandfit (salvianolic acid C).

As shown in Table 4, PTP1B possesses 12 natural products in the CDOCKER calculation procedure, and only salvianolic acid B could not dock to the PTP1B binding site. However, many compounds received very low scores compared with the reference molecule. Additionally, six compounds showed interaction with PTP1B in the Ligandfit calculation procedure. These six compounds also performed well in the CDOCKER calculation procedure. Compared to the initial ligand (527), almost all the hit compounds gained a little bit lower scores. Salvianolic acid C received the top docking score in both the two docking methods and shared part of the same hydrogen bond interactions and hydrophobic interactions with the key amino acid residues between the initial ligand (527). As shown in Figure 5, the key amino acid residues of PTP1B binding site were Tyr46, Phe182, Ser216, Cys215, Arg221, Ala217, Ile219, Ala27, and Val49. According to the CDOCKER calculation result, the interactions between the top hit (salvianolic acid C) and the binding site contained hydrogen bonds interactions with Arg221, Arg254, Gln262, Ala217, Ser216, Tyr20, and Agr24, electrostatic interactions with Arg221, Arg254, and Arg24, and hydrophobic interactions with Ala217, Cys215, Arg24, and Ala27. According to Ligandfit calculation result, the interactions between the top hit (salvianolic acid C) and the binding site contained hydrogen bond interactions with Arg24, Arg221, and Lys120, electrostatic interactions with Arg254 and Arg24, and hydrophobic interactions with Phe182, Val49, and Ala217. Additionally, rutin, quercitrin, salvianolic acid A, rosemary acid, and paeoniflorin also received high docking scores in at least one calculation procedure. And other compounds exhibited unsatisfactory performances in both docking processes.

Adenosine monophosphate-activated protein kinase (AMPK), which widely exists in the skeletal muscle, liver, pancreas, and adipose tissue, plays a key role that helps to maintain the intracellular balance of energy metabolism [34, 35]. When the ratio of AMP/ATP or ADP/ATP is increased, AMPK is activated to promote fatty acid oxidation. Accumulation of triglycerides (TG) or free fatty acids (FFAs) can cause and aggravate insulin resistance by activating protein kinase C (PKC) and protein phosphatase A2 (PPA2), which leads to PKC-mediated insulin receptor substrate (IRS) deactivation and PPA2-mediated AKT deactivation. Therefore, activation of AMPK can decrease insulin resistance by decreasing the accumulation of TG and FFAs.

**Figure 6:**
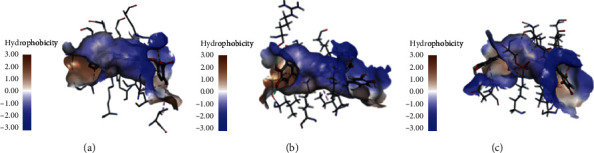
Interactions between AMPK binding site and ligands: (a) initial ligand; (b) top hit of CDOCKER (salvianolic acid C); (c) top hit of Ligandfit (salvianolic acid A).

AMPK is a heterotrimer composed of *α*, *β*, and *γ* subunits, in which the *α* subunit plays a catalytic role and the *β* and *γ* subunits play an important role in activity regulation and maintaining the stability of the trimer. AMPK can be activated indirectly and directly. Many direct activators, such as A-769662 and salicylic acid, activate AMPK by binding to the active site located in the cleft between the *β*-subunit central carbohydrate-binding domain and the *α*-subunit kinase domain [36]. As shown in Table 4, the selected representative compounds of active components in TZQ were evaluated for their docking abilities towards AMPK by the CDOCKER and Ligandfit methods. AMPK processed twelve natural products in the CDOCKER calculation procedure, and only salvianolic acid B could not dock to the binding site. Comparatively, seven compounds exhibited interaction with AMPK in the Ligandfit calculation procedure. The other six compounds that could not connect to AMPK in the Ligandfit calculation procedure also received very low docking scores as a result of the CDOCKER calculation procedure. Salvianolic acids A and C, quercetin, and rutin were ranked first in the two docking calculations. They shared part of the same hydrogen bond interactions and hydrophobic interactions with the key amino acid residues between the initial ligand (991). As shown in Figure 6, the key amino acid residues of AMPK binding site were Lys31, Lys29, Arg83, Ile46, Val113, Val24, Val81, Val113, and Val11. According to CDOCKER calculation result, the interactions between the top hit (salvianolic acid C) and the binding site contained hydrogen bonds interactions with Asn111, Asp20, and Sep108, electrostatic interactions with Lys29, Lys31, and Arg83, and hydrophobic interactions with Ile46, Val81, Val113, Lys29, Asp20, and Gly19. According to the Ligandfit calculation result, the interactions between the top hit (salvianolic acid A) and the binding site contained hydrogen bond interactions with Arg83, Arg107, Lys31, and Leu18, electrostatic interactions with Lys29, Arg83, and Lys31, and hydrophobic interactions with Val113, Ile46, Val81, and Val11.

Peroxisome proliferator-activated receptors (PPARs) belong to the nonsteroidal nuclear receptor superfamily and are ligand-activated transcription factors. There are three isoforms of PPARs that are encoded by different genes, and they exhibit a different ligand selectivity and biological specificity. PPAR*α* is a key target for lipid metabolism, and fibrate drugs targeting PPAR*α*, such as bezafibrate and fenofibrate, are indicated for triglyceride disorders. PPAR*δ* plays a potential role in lipid metabolism and cardiovascular diseases. PPAR*γ* can regulate glucose utilization and increase peripheral insulin sensitivity by promoting adipocyte differentiation and lipid storage. The use of PPAR*γ* agonists has become an effective and common treatment for type 2 diabetes mellitus. However, there are some inevitable side effects of single activation of PPAR*γ*, including peripheral edema, weight gain, elevated LDL cholesterol levels, and ultimately, an increased risk of cardiocerebrovascular disease. Therefore, in the development of antidiabetic drugs, more optimal research prospects exist for pan-PPAR agonists, which can simultaneously regulate glucose metabolism and lipid metabolism [37].

**Figure 7:**
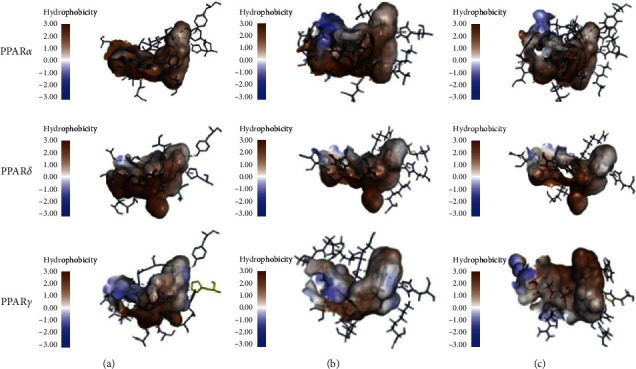
Interactions between PPARs binding site and ligands: (a) initial ligand; (b) top hit of CDOCKER; (c) top hit of Ligandfit.

The selected 13 representative compounds of active components in TZQ were evaluated for their docking abilities towards PPARs by the CDOCKER and Ligandfit methods, respectively (Table 5). As for PPAR*α*, all of the selected compounds exhibited varying degrees of interaction with PPAR*α* after the CDOCKER calculation procedure. However, nuciferine, 1-deoxynojirimycin, and danshensu could not dock to the binding site in the Ligandfit calculation procedure. These three compounds also received very low docking scores and were ranked at the bottom of the list in the CDOCKER calculation procedure. Salvianolic acids A, B, and C and rosemary acid from *Salvia miltiorrhiza*, rutin from *Morus alba*, paeoniflorin from *Paeonia lactiflora*, and quercetin from *Crataegus pinnatifida* exhibited high interaction with PPAR*α* in the two docking calculations. Meanwhile, as shown in Figure 7, the top hit compounds shared part of the same hydrogen bond interactions and hydrophobic interactions with the key amino acid residues between the initial ligand (13M). As for PPAR*δ*, salvianolic acids A and C and rutin received the highest scores in both docking calculations and shared part of the same hydrogen bond interactions and hydrophobic interactions with the key amino acid residues between the initial ligand (GW0, Figure 7). In addition, rosemary acid, paeoniflorin, tanshinone IIa, and quercetin obtained moderate scores in these two docking calculations. Nuciferine, 1-deoxynojirimycin, salvianolic acid B, and danshensu could not dock to the binding site in the Ligandfit calculation procedure. These four compounds also received very low docking scores in the CDOCKER calculation procedure, except for salvianolic acid B. As for PPAR*γ*, salvianolic acids A, B, and C and rutin received the top four scores in both docking calculations, whereas rosemary acid, paeoniflorin, and quercetin received moderate scores. 1-Deoxynojirimycin, danshensu, tanshinone IIa, cryptotanshinone, dihydrotanshinone I, and paeoniflorin could not dock to the binding site in the Ligandfit calculation procedure and also received very low docking scores in the CDOCKER calculation procedure. The top hits shared part of the same hydrogen bond interactions and hydrophobic interactions with the key amino acid residues between the initial ligand (544, Figure 7). Hence, it is suggested that many natural compounds in TZQ have potential pan-PPARs agonist activities. Salvianolic acids A and C from *Salvia miltiorrhiza* and rutin from *Morus alba* exhibited high interaction with all three isoforms. Rosemary acid and tanshinone IIa from *Salvia miltiorrhiza*, paeoniflorin from *Paeonia lactiflora*, and quercetin from *Crataegus pinnatifida* also exhibited potential activation activities against at least two isoforms.

**Figure 8:**
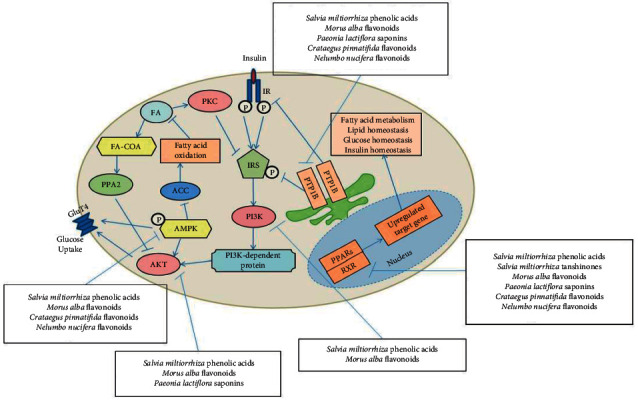
Summarized interactions between representative compounds and key signal proteins associated with IR.

Summarizing these all docking studies towards selected seven target proteins associated with insulin resistance, each representative compound of main active components in TZQ herbal formula exhibited different interaction abilities with those target proteins (Figure 8). *Salvia miltiorrhiza* phenolic acids, *Morus alba* flavonoids, and *Paeonia lactiflora* saponins exhibited strong docking abilities towards the PI3K/AKT pathway. *Salvia miltiorrhiza* phenolic acids and flavonoids from *Morus alba, Crataegus pinnatifida*, and *Nelumbo nucifera* exhibited strong docking abilities towards AMPK. *Salvia miltiorrhiza* phenolic acids, flavonoids from *Morus alba, Crataegus pinnatifida*, and *Nelumbo nucifera*, and *Paeonia lactiflora* saponins exhibited strong docking abilities towards PTP1B. And *Salvia miltiorrhiza* phenolic acids, *Salvia miltiorrhiza* tanshinone, *Morus alba* flavonoids, *Crataegus pinnatifida* flavonoids, and *Paeonia lactiflora* saponins exhibited strong docking abilities towards pan-PPARs. Alkaloids from *Nelumbo nucifera* and *Morus alba* did not get appropriate docking score in our study and may reduce IR from other target pathways. Among all the thirteen compounds, rutin from *Morus alba* and salvianolic acid B from *Salvia miltiorrhiza* exhibited high scores in both two docking calculation towards AKT and PPAR-*α*, salvianolic acid A from *Salvia miltiorrhiza* exhibited high scores in both two docking calculation towards PI3K, AKT, and PPAR-*δ*, salvianolic acid C from *Salvia miltiorrhiza* exhibited high scores in both two docking calculation towards PI3K, AKT, PTP1B, PPAR-*α*, and PPAR-*γ*, and paeoniflorin from *Nelumbo nucifera* exhibited high scores in both two docking calculation towards AKT.

## 3. Conclusion

Herein, a docking study of representative compounds of the main active components in TZQ with seven key signal proteins associated with insulin resistance was carried out using two different docking calculations. *Salvia miltiorrhiza* phenolic acids and *Morus alba* flavonoids may reduce IR by regulating all of the selected seven key proteins. *Crataegus pinnatifida* flavonoids and *Nelumbo nucifera* flavonoids may reduce IR by regulating PTP1B, AMPK, and pan-PPARs. *Nelumbo nucifera* saponins may reduce IR by regulating PTP1B, AMPK, and pan-PPARs. Tanshinone may reduce IR by regulating pan-PPARs. And *Nelumbo nucifera* alkaloids may reduce IR by regulating PPAR*γ*. These results have elucidated the multitarget mechanism that TZQ uses to regulate insulin resistance and have supported the rationality of our previous work to identify these components as the main active components. Meanwhile, these results have provided research directions for further in vivo/vitro pharmacological studies.

## Figures and Tables

**Table 1:** The plant origins and typical compounds

No.	Origin	Compound
1	Lotus leaf	Nuciferine
2	Mulberry leaf	Rutin
3	Mulberry leaf	1-Deoxynojirimycin
4	Salvia miltiorrhiza	Salvianolic acid A
5	Salvia miltiorrhiza	Salvianolic acid B
6	Salvia miltiorrhiza	Salvianolic acid C
7	Salvia miltiorrhiza	Danshensu
8	Salvia miltiorrhiza	Rosmarinic acid
9	Salvia miltiorrhiza	Tanshinone IIA
10	Salvia miltiorrhiza	Cryptotanshinone
11	Salvia miltiorrhiza	Dihydrotanshinone I
12	Hawthorn leaf	Quercitrin
13	Paeoniae rubra	Paeoniflorin

**Table 2:** RMSD values for the redocking studies.

PDB ID	PI3K (4WAF)	AKT (3OCB)	PTP1B (2QBP)	AMPK (4CFE)	PPAR-*α* (3VI8)	PPAR-*δ* (3TKM)	PPAR-*γ* (1K74)
RMSD (Ligandfit)	0.744	0.879	1.060	1.770	1.123	1.187	0.778
RMSD (CDOCKER)	0.660	0.844	0.743	1.403	1.266	1.116	0.487

**Table 3:** Docking scores of selected natural products with PI3K/AKT pathway.

No.	Compound	PI3K		AKT	
		CDOCKER (kcal/mol)	Ligandfit (kcal/mol)	CDOCKER (kcal/mol)	Ligandfit (kcal/mol)
1	3K6/XM1^Δ^	51.58	99.99	53.30	111.70
2	Nuciferine	33.65	—	33.47	69.27
3	Rutin	—	—	—	—
4	1-Deoxynojirimycin	27.02	—	40.57	41.87
5	Salvianolic acid A	62.08	95.40	62.30	132.11
6	Salvianolic acid B	88.52	61.04	62.96	137.50
7	Salvianolic acid C	73.10	110.94	68.05	136.21
8	Danshensu	35.79	—	34.51	65.42
9	Rosmarinic acid	45.80	34.49	43.55	109.42
10	Tanshinone IIA	34.06	60.44	33.34	61.32
11	Cryptotanshinone	33.70	59.30	30.48	67.51
12	Dihydrotanshinone I	30.89	65.08	29.57	62.22
13	Quercitrin	56.90	28.51	41.86	82.68
14	Paeoniflorin	—	63.02	51.57	112.15

^Δ^Initial ligand; 3K6 for PI3K (4WAF) and XM11 for AKT (3OCB).

**Table 4:** Docking scores of selected natural products with PTP1B and AMPK.

No.	Compound	PTP1B		AMPK	
		CDOCKER (kcal/mol)	Ligandfit (kcal/mol)	CDOCKER (kcal/mol)	Ligandfit (kcal/mol)
1	527/991^Δ^	102.50	90.50	64.08	100.61
2	Nuciferine	26.49	—	29.93	—
3	Rutin	86.52	55.79	78.84	67.84
4	1-Deoxynojirimycin	17.57	—	24.24	—
5	Salvianolic acid A	72.09	88.33	73.20	97.76
6	Salvianolic acid B	—	64.01	—	57.57
7	Salvianolic acid C	95.07	118.29	87.23	83.89
8	Danshensu	49.37	—	44.70	—
9	Rosmarinic acid	54.44	77.61	63.24	54.03
10	Tanshinone IIA	25.66	—	30.88	—
11	Cryptotanshinone	33.08	—	24.28	—
12	Dihydrotanshinone I	22.27	—	36.90	—
13	Quercitrin	83.52	—	81.89	55.11
14	Paeoniflorin	44.46	74.43	42.59	71.05

^Δ^Initial ligand; 527 for PTP1B (2QBP) and 991 for AMPK (4CFE).

**Table 5:** Docking scores of selected natural products with PPARs.

No.	Compound	PPAR*α*		PPAR*δ*		PPAR*γ*	
		CDOCKER (kcal/mol)	Ligandfit (kcal/mol)	CDOCKER (kcal/mol)	Ligandfit (kcal/mol)	CDOCKER (kcal/mol)	Ligandfit (kcal/mol)
1	13 M/GW0/544^Δ^	66.32	133.93	52.16	114.36	72.33	139.13
2	Nuciferine	38.01	—	19.99	—	43.04	37.02
3	Rutin	72.05	137.70	43.55	95.30	84.32	112.01
4	1-Deoxynojirimycin	20.07	—	20.45	—	21.17	—
5	Salvianolic acid A	65.91	135.36	65.44	113.77	70.32	122.67
6	Salvianolic acid B	77.34	155.10	43.67	—	61.24	116.77
7	Salvianolic acid C	68.31	137.90	45.09	100.83	89.51	136.18
8	Danshensu	30.96	—	23.60	—	35.06	—
9	Rosmarinic acid	48.40	114.79	27.61	75.29	57.83	72.71
10	Tanshinone IIA	37.60	73.90	18.06	63.29	31.17	—
11	Cryptotanshinone	35.54	71.71	18.90	59.27	31.79	—
12	Dihydrotanshinone I	37.15	75.01	18.61	51.19	36.58	—
13	Quercitrin	51.47	82.54	33.36	55.72	54.94	67.02
14	Paeoniflorin	44.79	103.09	32.11	70.69	43.97	—

^Δ^Initial ligand; 13M for PPAR*α* (3VI8), GW0 for PPAR*δ* (3TKM), and 544 for PPAR*γ* (1K74).

## Data Availability

Data and models used during the study are available from the corresponding author by request.

## Supplementary Materials

Supplementary Materials Table 1: the plant origins and structures of typical compounds.
