# Supplementary material for: Docking Studies on Potential Mechanisms for Decreasing Insulin Resistance by the Tangzhiqing Herbal Formula
Source: Evid Based Complement Alternat Med. 2020 Oct 9;2020:1057648. doi: 10.1155/2020/1057648 (PMC7568782; doi:10.1155/2020/1057648)
Supplement: Supplementary Materials — Table 1: the plant origins and structures of typical compounds. [file 1057648.f1.pdf]

Tab 1. The plant origins and structures of typical compounds

No.	Origin	Compound	Structure
1	Lotus leaf	nuciferine	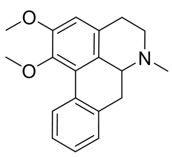
2	mulberry leaf	rutin	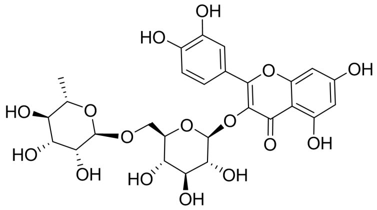
3	mulberry leaf	1-deoxynorijimycin	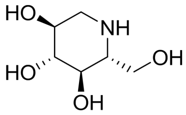
4	Salvia miltiorrhiza	Salvianolic acid A	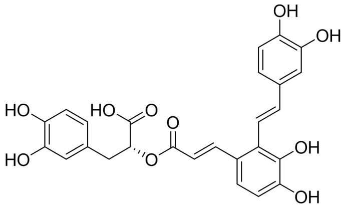
5	Salvia miltiorrhiza	Salvianolic acid B	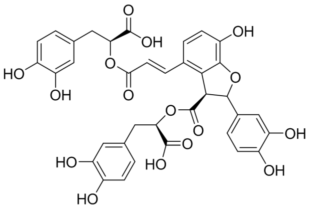
6	Salvia miltiorrhiza	Salvianolic acid C	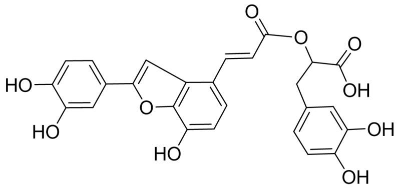
7	Salvia miltiorrhiza	Danshensu	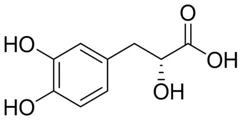

8	Salvia miltiorrhiza	Rosmarinic acid	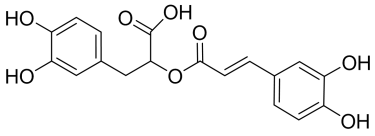
9	Salvia miltiorrhiza	Tanshinone IIA	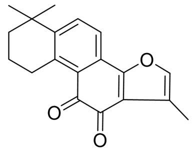
10	Salvia miltiorrhiza	Cryptotanshinone	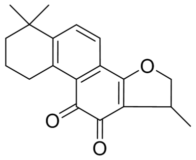
11	Salvia miltiorrhiza	Dihydrotanshinone I	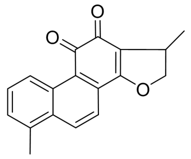
12	hawthorn leaf	Quercitrin	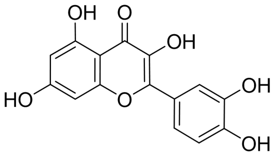
13	Paeoniae Rubra	paeoniflorin	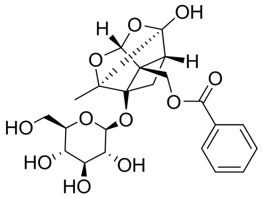
